# Introduction and History of the Chemistry of Nucleic Acids Therapeutics

**DOI:** 10.1007/978-1-0716-2010-6_1

**Published:** 2022-01-01

**Authors:** Michael J. Gait, Sudhir Agrawal

**Keywords:** Antisense, siRNA, Nucleic acid therapeutics, Oligonucleotides, Toll-like receptors, Pattern recognition receptors, Gapmer, Splice switching

## Abstract

This introduction charts the history of the development of the major chemical modifications that have influenced the development of nucleic acids therapeutics focusing in particular on antisense oligonucleotide analogues carrying modifications in the backbone and sugar. Brief mention is made of siRNA development and other applications that have by and large utilized the same modifications. We also point out the pitfalls of the use of nucleic acids as drugs, such as their unwanted interactions with pattern recognition receptors, which can be mitigated by chemical modification or used as immunotherapeutic agents.

## 1 Introduction to Synthetic Antisense Oligonucleotides and siRNA

Oligonucleotides are short single-stranded sections of DNA or RNA that contain 2′-deoxyribo-nucleosides or ribo-nucleosides, respectively, which are linked by 3′–5′phosphodiester linkages (Fig. 1a). Antisense oligonucleotides are those that are complementary to a section of naturally occurring RNA, such as an mRNA or a viral RNA, to form Watson–Crick base pairs and to thus inhibit a biological function of that RNA. Zamecnik and Stephenson pioneered this concept in 1978 by utilizing antisense oligodeoxyribonucleotides (ODNs) to bind and inhibit the replication of Rous sarcoma virus (RSV) RNA [1]. This work followed much earlier (1969) studies of De Clercq et al. on interferon induction by synthetic polynucleotides and their phosphorothioate analogues [2] and together these early studies heralded the new field of nucleic acids therapeutics that began to accelerate in the mid to late 1980s.

Many further chemistry developments since then in the use of synthetic oligonucleotide analogues, as outlined below, as well as advances in molecular biology, such as in the newer fields of short interfering RNA (siRNA) and non-coding RNAs, such as micro-RNA (miRNA), have led to the widespread and convenient use of synthetic oligonucleotides as antisense and siRNA reagents for gene ablation or targeting of non-coding RNA, as well as their use in animals and in humans leading to the approval of 12 drugs to date. In this chapter we outline the history of oligonucleotide chemistry in antisense and siRNA that has led to preclinical studies that have guided their development with drug-like properties and hence clinical trials (Fig. 2). We go on to discuss the development of the principles of widely used antisense gapmers and siRNAs as well as their immune responses by triggering pattern recognition receptors (PRRs) and how such activities can be controlled or harnessed for use as drugs (Fig. 3). Readers are also referred to a recent book edited by us on recent Advances in Therapeutic Nucleic Acids [3].

## 2 Oligonucleotide Synthesis

Early work in the 1960s and 1970s on phosphodiester and phosphotriester chemistry for the synthesis of ODNs required armies of nucleic acids chemists for painstaking oligonucleotide synthesis in solution phase that took months to years for each synthesis (for example [4]). The revolution for molecular biologists came in the late 1970s and early 1980s with the development of solid-phase ODN synthesis first suggested by Letsinger and Mahadevan in 1965 [5] and later developed into working methods in the laboratories of Gait [6–8] and Itakura [9]. These methods were then superseded by the more efficient phosphoramidite chemistry of Caruthers and colleagues [10], which went on to be automated, such as by Applied Biosystems and other companies. The rapid and automated synthesis allowed molecular biologists to obtain synthetic ODNs readily for biological purposes such as for sequencing, cloning, and gene synthesis. The history of oligonucleotide synthesis chemistry has been reviewed [11, 12]. Today standard and modified ODNs can be obtained rapidly and efficiently on a small to large scale through highly automated solid-phase DNA oligonucleotide synthesis for many biological and diagnostic purposes.

Oligoribonucleotide synthesis is also now well established for the synthesis of siRNA or for aptameric RNAs. Currently, several oligonucleotide manufacturing plants are operational to produce oligonucleotides under GMP conditions.

## 3 Synthetic Oligonucleotide Analogues in Antisense and siRNA

The early work of Zamecnik and colleagues utilized RSV since this was the only viral RNA sequenced at this time. Zamecnik noticed that both ends of the linear RNA genome bore the same primary sequence in the same polarity and that DNA might be synthesized from the RNA by reverse transcription via circularization of the 5′-end with the 3′-end through base pairing. Zamecnik and Stevenson were able to block this circularization by use of a synthetic ODN via hybridization with the 3′-end of the viral RNA. By use of a cell-free system, translation of the RSV mRNA was impaired, thus leading to inhibition of viral replication. This work was the birth of the antisense concept [1, 13].

Further progress in the antisense field awaited the mid to late 1980s for the availability of genomic DNA (or RNA) sequences for antisense targeting as well as the new automated methods of oligonucleotide synthesis as described above. There was also some scepticism regarding the stability and eukaryotic cell entry ability of unmodified oligonucleotides. Nevertheless Zamecnik and Gallo were able to employ unmodified antisense ODNs to inhibit human immunodeficiency virus 1 (HIV-1) replication [13] and to suppress expression of HIV-1 related markers [14]. Cellular uptake of the ODN was not a limiting factor since experiments were carried out in primary human cells and non-targeted control oligonucleotides showed minimal inhibition of HIV-1 replication, thus demonstrating sequence-specificity. This paper reignited the therapeutic potential of the antisense approach.

The next step was to provide drug-like properties to the unmodified antisense ODNs through chemical modifications. In this context the key consideration was to provide nucleolytic stability to antisense ODNs without affecting their hybridization and affinities with the RNA target. Not much was known at the time about the in vivo characteristics of unmodified ODN, or any modified ODNs, which could have guided the study of potential modifications. The first step was to see if modifications of the internucleotide linkages would provide nucleolytic stability to ODNs, while preserving the hybridization affinity to the target RNA. Later on, discovery of PRRs further provided insights into recognition of pathogen associated molecular patterns (PAMPs) of nucleic acids, and how sequence of antisense and nucleic acid-based therapeutic approaches could affect the mechanism of action (Fig. 3).

### 3.1 Backbone Analogues

#### 3.1.1 Phosphorothioates

The antisense field took inspiration from the very early work of De Clerq, Eckstein, and Merigan [2] where phosphorothioate (PS) modifications were studied in homopolynucleotides as stabilizing agents. By the mid-1980s new chemical synthesis methods for the PS linkage in ODNs became available [15]. Here, a simple sulfur atom replaces a non-bridging oxygen atom (Fig. 1b). However, standard automated synthesis, which in the phosphoramidite method involves treatment with a sulfurizing agent in place of oxidation by iodine, produced a mixture of diastereomeric oligonucleotide products (*R*p and *S*p) (Fig. 1c, d) and thus there was found to be a lower binding affinity to target RNA compared to phosphodiesters. However, PS-linked ODNs are much more resistant to nuclease degradation than phosphodiesters. Optimization of the synthesis methodology allowed the synthesis of milligram quantities of PS-ODNs for use in cell and in vivo experiments.

Early studies showed dose-dependent inhibition of viral replication and antiviral activity in HIV-1 infected cells by use of antisense PS-ODNs targeted to several regions of HIV-1 mRNA [16, 17]. Surprisingly, homopolymers were also effective and antiviral activity depended on the base composition, suggesting that PS-ODNs also had off-target activity. Longer PS-ODNs were more effective than shorter ones and cellular uptake was efficient in primary human cells without a carrier. In addition, antisense PS-ODNs showed potent and durable inhibition of HIV-1 replication in chronically HIV-1 infected cells [18–20]. Soon after, studies with PS-ODN targeted to influenza virus showed inhibition of virus replication [21]. Based on these early studies and promise, PS-ODNs became the choice for first-generation antisense agents.

Following these studies, work on antisense was pursued in many laboratories around the world for a broad range of applications [22–24]. In addition, several new companies were founded to advance therapeutic applications of antisense, such as Gilead Sciences, Isis Pharmaceuticals (now Ionis Pharmaceuticals), Hybridon (now Idera Pharmaceuticals), and others. Numerous reports appeared on the use of antisense PS-ODNs to target viruses [25, 26], oncogenes [24, 27], and kinases [27, 28], etc. Cellular uptake of PS-ODNs in transformed cells in culture was found to be poor but could be improved substantially by use of cationic lipid carriers. It was also shown that an antisense PS-ODN bound to its target RNA engaged RNase H [19, 20] to excise the RNA strand and this was therefore likely to be the mode of action and not steric block inhibition of translation as observed with other modified ODNs [29, 30]. Nevertheless RNase H cleavage activity was poorer than for a PO-ASO [20].

The first in vivo study in mice of a systemically delivered PS-ODN showed that plasma half-life was very short but that there was a broad tissue disposition with most delivered to liver and kidneys and the lowest amounts in the brain [31]. The PS-ODN was stable in tissues for several days and excreted primarily in urine in degraded form mostly through exonuclease cleavage from the 3′-end. Chemical modifications at the 3′-end increased the stability [32, 33]. The PS-ODN was bound by serum proteins, which increased the plasma half-life and improved tissue disposition [34, 35].

Antisense PS-ODNs showed very potent activity in animal models of viral diseases and cancer [36, 37]. However, in some cases a control PS-ODN also showed some activity, leading to the possibility of off-target effects [38]. For example, a PS-ODN targeted to human papillomavirus inhibited papillomavirus-induced growth of implanted human foreskin in a mouse xenograft model but unexpectedly was also active in a mouse cytomegalovirus (CMV) model [39]. Studies in immune-compromised mice showed that the effect of the PS-ODN was largely due to immune activation of the host. Sequence-dependent immune stimulation was confirmed during non-clinical safety evaluations of drug candidates. Repeated systemic administration of PS-ODN candidates in mice and rats caused inflammation, splenomegaly, and histological changes in multiple organs [40, 41]. Further in non-human primates, bolus administration of the first antisense PS-ODN (GEM91) led to severe hemodynamic changes due to activation of the alternative complement pathway [42]. Stimulation of the alternative pathway complement activation cascade became the first documented off-target effect, which was due to a plasma concentration effect of the poly-anionic nature of PS-ODNs. It could be mitigated by subcutaneous administration or by slow intravenous infusion. Thereafter, the US Federal Drug Agency (FDA) published guidelines and required the use of non-human primates for non-clinical safety studies of all oligonucleotide drugs [43].

In the 1990s a number of antisense PS-ODN clinical candidates were advanced to human trials [44] through intravenous infusion, intravitreal or subcutaneous delivery [45–47]. Humans showed similar pharmacokinetics and excretion data to those of non-human primates [48, 49]. However, most clinical studies were discontinued due to the lack of activity or a poor therapeutic index [50]. For example, the subcutaneous administration of GEM91 in humans caused flu-like symptoms, swelling of the draining lymph nodes, prolongation of activated partial thromboplastin time (aPTT), and thrombocytopenia [29]. Rather than suppression of HIV-1, HIV-1 RNA levels were increased in blood [51]. However, intravenous delivery had minimal effect on these parameters. There were a few reports of immunostimulatory properties of DNA/ODNs containing CG nucleotides [52, 53]. It only became clear much later that PS-ODNs containing an unmethylated CpG motif activated the immune responses by binding to Toll-like receptor 9 (TLR9), an innate immune receptor present in immune cells that recognizes DNA containing CpG dinucleotide motifs [54]. It became clear that the flu-like symptoms and injection site reactions seen with most of the PS-ODN drug candidates in clinical trials, such as the clinically approved drug fomivirsen, administered intra-vitreally to treat AIDS-related CMV-induced retinitis, contained a CpG motif [55]. Thus, the true mechanism of action of this first-generation drug, now no longer marketed, remains unclear. Altogether, preclinical and clinical studies have provided important insights into the properties of PS-ODNs and its use as drugs [35, 56, 57].

Also of debate for some time has been whether the presence of a mixture of *R*p and *S*p diastereoisomers in the synthetic PS-ODNs (Fig. 1c, d) bears any influence on their biological properties. For example, a 20-mer would have 2^19^ isomers. The stereospecificity of enzymes that act on nucleoside phosphates was well known from early work of Eckstein (reviewed in [58]). Since PS-ODN interacting enzymes, such as nucleases, also utilize only a single diasterioisomeric isomer [59], it was plausible that there might be a significant biological effect in cells of utilizing mixed PS diastereomers in antisense PS-ODNs. Testing of this only awaited the solid-phase synthesis of stereo-enriched and stereo-pure PS-ODNs. This became possible through pioneering work of Stec and later by use of nucleoside bicyclic oxazaphospholidinium synthons [15, 60]. It is now known that binding strength and recognition by RNase H is generally higher for antisense oligonucleotides containing *R*p linkages but depends crucially on the placement of these with respect to *S*p linkages and overall stereospecific PS-ODNs have had limited therapeutic utility [61]. Recently certain stereo-pure antisense oligonucleotides were shown to have improved activity in cell culture and in vivo [62] but the therapeutic significance of such stereospecificity is currently hotly disputed [63]. Even more recently, the clinical development of a stereo-pure PS-ODN, WVEN-531 targeted to DMD has been discontinued due to lack of clinical activity [64]. Furthermore, dosing of this antisense ODN also led to transient increases in complement factors and C-reactive protein [65].

#### 3.1.2 Charge-Neutral Analogues

Two phosphate-containing, charge-neutral oligonucleotide analogues that were particularly used in early antisense studies are the methylphosphonate [66] (Fig. 1e) and the phosphoramidate linkages [67] (Fig. 1f). They both consist of a mixture of diastereoisomers. Methylphosphonate ODNs (MP-ODNs) are stable at physiological pH and are resistant to nucleolytic degradation but are less strongly bound to target RNA compared to PO-ODNs [66, 68]. MP-ODNs targeted to HIV-1 showed dose-dependent inhibition of HIV-1 replication [16], but they are less active than PS-ODNs due to their lack of RNase H activation [19] but instead inhibit protein translation, which is generally a weaker activity in cells. Limited in vivo studies with a MP-ODN showed that while this modification is very resistant to nucleolytic degradation, due to poor protein binding, there was a very poor in vivo disposition and the majority of the administered ODN was eliminated in urine rapidly (Agrawal, unpublished data). In addition, longer MP-ODNs, which bind more strongly to RNA and which are therefore more potent, are poorly soluble under physiological conditions and thus have not been advanced toward clinical trials. By contrast, a 13-mer antisense oligonucleotide containing all phosphoramidate linkages is more soluble. An anticancer agent (GRN163) inhibits the enzyme telomerase [67] and did get into a clinical trial, however, clinical development was discontinued due to lack of clinical activity. In early work, phosphoramidate-linked antisense oligonucleotides (Fig. 1g, h) targeted to HIV-1 showed similar results in cell-based assays to an MP-ODN and were not pursued [16].

Phosphorodiamidate morpholino oligonucleotides (PMOs) are also charge-neutral but here a morpholino ring replaces the sugar unit (Fig. 1i) [69]. PMOs inhibit translation by a steric block mechanism [70] as they are not recognized by RNase H. They are completely resistant to nucleases but are not taken up well by cells and thus require very high doses for in vivo delivery. They were found to be strong antiviral agents, for example, against Ebola, Marburg, and Chikungunya viruses [71]. Three exon skipping PMO drugs, eteplirsen, golodirsen, and vitolarsen designed to induce alternative splicing and restore the reading frame of mutant dystrophin in patients with Duchenne muscular dystrophy (DMD) [72] have been approved but requires the use of high doses (50 mg/kg or higher). Its therapeutic effectiveness, based on biomarkers, is limited [73], but it is a safe drug at the therapeutic dose. PMO and other chemistries used in exon skipping and other steric block activities have been reviewed [74].

Another initially highly promising, charge-neutral analogue are peptide nucleic acids (PNA), where the sugar-phosphate backbone is replaced by aminoethylglycine units linked by amide bonds (Fig. 1j) [75]. PNA binds strongly to target RNA and, like PMO, they are also completely resistant to degradation by nucleases as well as proteases. Also similar to PMO, duplexes with RNA are not recognized by RNase H and thus PNA acts by a steric block mechanism. Antisense PNAs have been broadly studied as anticancer [76, 77], antiviral [78, 79], and antibacterial agents [80, 81] as well as inhibitors of micro-RNAs [82]. However, once again very high doses are needed in in vivo applications, due to poor cellular uptake and unfavorable pharmacokinetics. Poor in vivo biodistribution is a likely reason for why antisense PNAs have not to date found utility as clinical candidates.

### 3.2 Sugar Analogues

It has been long established that an RNA-RNA duplex is much stronger than that of DNA-RNA. However, RNA (Fig. 4a) is highly unstable to ribonucleases. Phosphorothioate analogues (PS-ORN) showed an increased affinity to target RNA but they were found to have lower potency as compared to PS-ODNs, probably since RNA-RNA duplexes lack RNase H activation ability [35].

#### 3.2.1 2′-O-Alkyl Sugars

The first sugar analogues to find utility in antisense oligonucleotides are the naturally occurring 2′-*O*-methylribonucleosides (Fig. 4b) first synthesized by the Ohtsuka laboratory [83]. The phosphoramidites of 2′-*O*-methylribonucleosdes suitable for solid-phase synthesis became available commercially in the early 1990s [29]. Studies with 2′-*O*-methyloligoribonucleotide phosphorothioates (2′-OMe PS-ORN) showed enhanced stability to nucleases as compared to PS-ORN and showed a higher affinity to target [84]. However, they also showed lower antisense activity compared to PS-ODNs, demonstrating that activation of RNase H was key for this activity [30, 51, 85, 86]. Since then, very many additional 2′-*O*-alkyl analogues have been synthesized and tested in antisense oligonucleotides (ONs), predominantly in gapmer studies (see below) to allow recognition by RNase H. From these studies 2′-*O*-methoxyethylribonucleoside (2′-MOE) (Fig. 4c) has been employed widely in clinical gapmer candidates (Chapters 3 and 4 of Agrawal and Gait [3]).

As described earlier, dose-dependent activation of complement and prolongation of aPTT were found to be unwanted side effects of PS-ODNs. These effects as well as strong binding to serum proteins were thought to be due to the poly-anionic nature of the PS linkage. However, there were found to be significantly less side effects when PS-ORN or 2′-OMe-PS-ASO where used, suggesting that the poly-anionic nature of the PS backbone in PS-ORN and PS 2′-OMe is not as pronounced when placed in the context of an RNA or RNA-like sugar conformation [40, 85–87]. This became crucial to their use in later gapmer antisense studies.

Uniformly 2′-*O*-alkyl modified PS-RNA has also found very high therapeutic use in splice switching (exon skipping or exon inclusion) and other steric blocking applications due to their high binding strength to nuclear pre-mRNA [88, 89]. However, the exon skipping 2′-OMe PS-ORN antisense drisapersen drug candidate failed to show clinical benefit in patients with DMD and also caused significant adverse side effects and was, therefore, not approved for clinical use [90]. By contrast, the 18-mer 2′-MOE phosphorothioate (2′-MOE PS-ORN, nusinersen), which redirects the splicing of the SMN-2 gene to generate active SMN protein (exon inclusion), administered intrathecally only few times a year was approved by the FDA for the treatment of spinal muscular atrophy (SMA) [91, 92]. The thrombocytopenia and renal toxicity observed with the use of drisapersen could be largely due to the need for repeated subcutaneous dosages of 2′-OMe PS-ORN, which is very stable to nucleolytic degradation and therefore may accumulate in tissues due to its long half-life and potentially interact with PRRs. Nusinersen, a 2′-MOE PS-ORN, is also quite stable to nucleolytic degradation, but its intrathecal administration and need for infrequent and lower doses minimizes the impact of tissue accumulation and avoids the need for passage into the brain and spinal cord from the circulation.

#### 3.2.2 Locked/Bridged Nucleic Acids

A major step forward in the design of antisense ONs was the development in the laboratories of Wengel and also of Imanishi of bicyclic sugar analogues known as locked or bridged nucleic acids (LNA/BNA). Here the conformational flexibility of nucleotides is significantly reduced by linkage of the 2′-oxygen atom to the 4′-carbon atom in the ribose ring (Fig. 4d). This results in a significant increase in the binding affinity of ONs to complementary RNA targets with an increase in the melting temperature of 2–8 °C per residue [93]. Unfortunately, LNA oligomers of 8 units or longer tend to self-aggregate. Therefore they became more useful as mixmers with 2′-deoxynucleotides and here miravirsen, the first microRNA-targeting drug, which acts by sterically blocking microRNA-122, highly expressed in liver, was developed for the treatment of hepatitis C virus infection, a debilitating liver disease [94]. Unfortunately, this drug’s clinical development was discontinued because of *safety* issues. LNA has also been used as mixmers with 2′-*O*Me nucleotides targeting various RNAs in cells (e.g. [93]) and has also found utility in the flanking sequences of gapmers. This had the effect also of modulating the binding strength of the ON and increasing the specificity of the interaction (reviewed in Chapter 3 of Agrawal and Gait [3]).

Another bicyclic analogue that became useful is 2′-*O*,4′-C-ethylene linked nucleic acid (ENA) [95] (Fig. 4e). In a recent study an antisense ON DS-5141, containing segments of 2′-OMe PS-RNA and ENA, showed good activity in an *mdx* mouse model of DMD and a phase 1/2 clinical trial was carried out in Japan [96]. A further analogue useful in steric block applications is tricyclo-DNA (tcDNA) [97] (Fig. 4f). However, perhaps the most important bicyclic derivative of LNA that has found considerable therapeutic utility is the methylated analogue known as “constrained Ethyl” (cET), which is being employed in shorter gapmers [98] (Fig. 4g) and being evaluated in preclinical and clinical studies by Ionis Pharmaceuticals Inc. All these types of bridged nucleic acids have shown very strong affinity to target RNA and increased nucleolytic stability, but none of them are substrates for RNase H. Thus, these types of bicyclic sugar analogues are mostly used in steric block/splicing modulation approaches and in the flanking sequences of gapmers.

### 3.3 Heterocyclic Base Analogues

In early antisense studies it was thought that increased antisense activity might be achievable by improving the affinity of an ODN to target RNA through modification of the heterocyclic bases, for example, by adding an extra hydrogen bond in the base pairing between an ODN and its RNA target or by increasing the base stacking potential in a DNA–RNA duplex. Chemically this was simplest through modifications in the pyrimidine rings, for example, by modifications at positions C-2, C-4, C-5 or at C-6, and many of these base analogues were incorporated into antisense ODNs. However, few of these proved to be of significant value. Incorporation of modified purines generally resulted in a reduced binding affinity of an antisense ODN. Perhaps the most useful study of antisense activity was of incorporation of various heterocyclic bases in ODNs including the increased base stacking analogues C-5 propynyl and 5-methyl cytosine (5-MeC) and the increased hydrogen-bonding analogues phenoxazine, and G-clamp. These studies showed that the increased hydrogen-bonding analogue G-clamp had potent dose-dependent antisense activity [99]. Unfortunately, these antisense ODNs containing G-clamps were found to be highly toxic in in vivo studies. Currently, the only significantly used nucleoside base analogue in antisense ODNs is 5-methyl-2′-deoxycytidine (5-MedC) [100]. This methylated base analogue is used mainly to mitigate immune activation in CpG dinucleotide sequences rather than for changing binding strength [101].

## 4 RNase H Active Gapmer Chemistry for Use as Drugs

Early studies conducted with various modified ODNs, ORNs, and 2′-substituted ORNs as antisense agents provided great insights into what is important for providing drug-like properties to antisense oligonucleotides [3]. Studies with PS-ODNs showed that increased nucleolytic stability and activation of RNase H were key. However, polyanion-related side effects and sequence-dependent immune activation were limiting factors in their broad applicability [50, 57]. Studies with MP-ODNs showed that polyanion-related side effects could be completely mitigated (Agrawal, unpublished data), and had significant nucleolytic stability. However, with lower affinity and lack of RNase H activation, there was a loss in antisense potency [16]. These observations led to the concept of combining desirable properties of the two modified ODNs to provide drug-like properties to antisense oligonucleotides [29]. The first studies were carried out with antisense containing segments of PS-ODN and PM-ODN or PN-ODN referred to as mixed backbone antisense ONs. These antisense designs showed increased nucleolytic stability and RNase H activation [19, 40]. However, reduced affinity limited their potency. Further insight was obtained from in vivo studies in which a mixed backbone ON containing PS-ODN and PM-ODN showed wide tissue disposition and increased stability and longer half-life in tissues [102].

This led Agrawal and colleagues to design antisense oligonucleotides in which the segments of PS-ODN and 2′-substituted PS-ORN were combined at the appropriate positions [29, 84, 85]. These types of antisense oligonucleotides were referred to as Hybrid ONs, now commonly referred to as gapmers (Fig. 5). In the original design of antisense, a segment of PS-DNA was placed in the middle and segments of 2′-*O*-alkyl PS-ORN or a combination of PO- and PS-linkages were placed at both 3′- and 5′-ends [29, 84, 85, 87, 103]. This design of antisense combined the desirable properties of PS-DNA and 2′-*O*-alkyl PS-ORN, and provided increased affinity to targeted RNA, activation of RNase H, increased nucleolytic stability, and reduced polyanion-related side effects. Furthermore, inflammatory responses were also reduced [35, 40, 56]. In vivo administration in mice showed similar plasma half-life and tissue disposition similar to that observed with PS-ODNs, and with increased in vivo stability and retention in tissues [104]. Also, due to increased in vivo stability, oral and rectal delivery of gapmer antisense was possible [34]. It was postulated that the increased stability and in vivo persistence may allow less frequent dosing to obtain therapeutic benefits.

There was also a concern that increased retention of gapmer antisense in tissues may lead to tissue build up following repeated dosing, which would induce local inflammatory responses and side effects, thereby limiting its therapeutic potential.

Other configurations of gapmer antisense were also evaluated, including the configuration in which a segment of 2′-*O*-alkyl PS-ORN was placed in the center and segments of PS-DNA were placed at both 3′- and 5′-ends. This design of gapmer antisense showed increased potency compared to PS-DNA and reduced polyanion-related side effects. In general, the specificity of RNase H mediated cleavage and its efficiency and excision sites were dependent on the position of the PS-DNA in gapmer antisense ONs [105].

Based on these encouraging results, gapmer antisense became the choice for second-generation antisense agents. In 2001, a licensing agreement between the companies allowed the technology to be widely available [44]. Over the years, studies have been carried out to establish the optimal size of the window of a central PS-DNA segment [105]. Similarly, studies have been carried out to optimize the size of the modified ORN wings at both 3′- and 5′-ends. In the wings of the gapmer antisense, various modified ORNs have been incorporated and evaluated (*see* Chapter 3 of Agrawal and Gait [3]). To date, most promising results have been obtained with gapmer antisense containing segments of 2′-*O*-methyl or 2′-*O*-methoxyethyl at both 3′- and 5′-ends. Over 30 gapmer antisense drug candidates containing 2′-*O*-methoxyethyl or LNA segments have been advanced to clinical evaluations following systemic delivery. To date, three candidates have been approved for clinical use. These include inotersen [106], volanesorsen [107], and mipomersen [108]. Clinical development of several gapmer antisense drug candidates have been discontinued, due to lack of clinical activity and or safety signals. These include ISIS-FXIRx, ISIS-EIF4ERx, ATL1103, ATL1102, ISIS-GCGRRx, ISIS-PTBRx, ISIS-APOARx, ISIS-SOD1Rx, ISIS-FGFR4Rx, ISIS-405879, OGX-011, OGX-427, LY2181308, ATL1103, ATL1102, etc.

As discussed above, many of the bicyclic sugar analogues including locked/bridged nucleic acids have been studied as antisense agents. These analogues have also been studied as part of the wings in gapmer antisense. These include LNA (Fig. 2d) [109], constrained ethyl 2′-4′ bridged nucleic acid (cEt) (Fig. 2g) [110], anhydrohexitol [91], fluorocyclohexenyl (F-CeNA) [111], altritol nucleic acids [112], and tricyclo-DNA (tcDNA) (Fig. 2f) [97]. These modifications provide very high affinity and have allowed the length of the gapmer antisense to be reduced. A few of these shorter gapmer ASOs are being employed to achieve allele specific knockdown [113]. While the use of shorter antisense may be cost effective, it increases the possibility of off-target effects by binding to non-targeted RNA [114, 115]. Also selected LNA and cET ASOs have been associated with liver toxicity [116, 117].

Several gapmer antisense drug candidates employing LNA in the wings have advanced toward the clinic but development of most of these candidates have been discontinued, primarily due to safety issues and lack of therapeutic index.

Other modifications in the gapmer antisense studies include 2′-deoxy-2′-fluoro-beta-D-arabinonucleic acid (FANA) [118], 3′-fluorohexitolnucleic acid (FHNA) [119], 2′-thiothymine, 5-modified pyrimidine bases, etc. These studies are limited to pre-clinical evaluations.

## 5 siRNA Chemistry for Use as Drugs

The lessons learned in the development of antisense ONs have allowed the development of siRNA therapeutics to be speeded up. siRNAs have a well-defined structure: a short double stranded RNA of 20–25 base pairs with phosphorylated 5′-ends and hydroxylated 3′-ends and also usually containing two 3′-overhanging nucleotides, although blunt ends are sometimes used. While the key requirement is to provide nucleolytic stability to a siRNA candidate, it requires an understanding of the function of each strand. One strand is called the passenger strand and the other is the active component and is called the antisense or guide strand. It is the guide strand that is incorporated into the enzyme complex called RISC in order to be directed to cleave the target RNA strand, while the passenger strand is displaced.

Studies of various chemical modifications in antisense and their impact on providing drug-like properties have allowed the use of some of these modifications in development siRNA therapeutics. These include PS-linkages, various modified ribose sugars such as 2′-*O*-methyl, 2′-fluoro 2′-deoxy (2′-F), LNA as well as the sugar ring-opened analogues unlocked nucleic acid (UNA), and glycol nucleic acid (GNA). In siRNA chemical modifications are introduced strategically to provide nucleolytic stability. In addition the passenger strand is usually heavily modified in order to block passenger strand entry into RISC, while to promote RISC loading of the guide strand only light modification is used, such as 2′-F replacement of 2′-OH groups in pyrimidines. At the same time modifications must not be placed centrally in the guide strand so as to block RISC-associated cleavage of the target RNA. The exact locations of such modifications in guide and passenger strands are generally closely guarded secrets by reagent suppliers. In addition, the 5′-phosphate of a siRNA guide strand is essential for recognition by RISC. Phosphatase-resistant analogues of the 5′-end phosphate have been shown to improve the in vivo efficacy and are used in clinical candidates [120].

In siRNA candidates, chemical modifications provide nucleolytic stability, however, delivery to a desired tissue or cell type requires use of carrier or conjugation with delivery moieties [121]. To date, two main delivery platforms—ionisable lipid nanoparticles (iLNPs) and trivalent N-acetylgalactosamine (GalNAc) conjugates—have been employed for delivery to liver hepatocytes. To date, two siRNA drugs have been approved for clinical use patisiran, which uses lipid delivery, and givosiran, which uses a GalNAc conjugation. Third siRNA drug candidate, inclisiran, a GalNac conjugate has shown positive results in phase 3 trial [122]. Details of these structure activity relationship studies have been discussed in two chapters from a previous book [123, 124].

## 6 Immune Responses to Nucleic Acids

Over the last five decades there have been several reports on observations that certain nucleic acid sequences showed immune stimulatory properties [52, 125, 126]. In the mid-1990s subcutaneous administration of an antisense PS-ODN targeted to HIV-1 (GEM91) in HIV-1 infected individuals caused flu-like symptoms and systemic immune responses [51]. This observation alone could not be explained until the discovery of PRRs. These receptors are part of the immune system and PAMPs and host-derived damage-associated molecular patterns (DAMPs). These PRRs play an essential role in establishing antiviral and antibacterial responses by recognizing PAMPs. However, PRRs could also induce development of autoimmune and inflammatory diseases by recognizing DAMPs [127, 128].

PAMPs are highly conserved motifs in pathogens, such as bacteria and viruses. There are several PRRs that are known to recognize motifs, sequences, and patterns of nucleic acids and induce receptor-mediated immune responses. These include members of Toll-like receptors (TLRs), of which four TLRs respond to nucleic acids. TLR3, TLR7/8, and TLR9 recognize double-stranded RNA (dsRNA), single-stranded RNA with certain sequence composition and modified bases (ssRNA), and DNA containing unmethylated CpG sequences (CpG DNA), respectively (Chapter 13 of Agrawal and Gait [3]; [129]). These TLRs are localized in endosomes and expressed on various cell types. The type of immune response induced varies dependent on the receptor and the nature of the nucleic acid [130]. In addition to TLRs, additional receptors are present in the cytoplasm known to recognize nucleic acid-based PAMPs. These include retinoic acid-inducible gene-I (RIG-I), melanoma-associated gene-5 (MDA-5), absent in melanoma 2 (AIM2), cyclic-AMP synthase (cGAS), and stimulator of the interferon gene (STING) (Chapter 13 of Agrawal and Gait [3]).

Following the discovery of TLR9, it became clear that immune activation observed with administration of GEM91 was due to the presence of unmethylated CpG dinucleotides in the antisense sequence [131]. This also provided insights into many of the preclinical studies that the chosen antisense may be exerting antiviral or anticancer activity due to immune activation and not by an antisense mechanism [51, 57]. Interestingly, most of the antisense PS-ODNs in clinical development contained unmethylated CpG motifs, raising questions on the intended mechanism of action [51, 57]. Clinical development of all these antisense drug candidates was discontinued due to lack of activity but also due to safety signals. Similar observations have been made with a few initial siRNA candidates and once again mechanisms of action have been correlated with activation of immune responses [132, 133].

The discovery of PRRs has provided key insights into many of the observations made with use of PS-ODN antisense. For example, TLR9 is a receptor for synthetic ODNs containing unmethylated CpG motifs [131]. Activation of TLR9 leads to induction of Th1 type immune responses in mice, primates, and in humans (Chapter 14 of Agrawal and Gait [3]). Inductions of Th1 type immune responses, which include type interferon (IFN) and inter-leukin 12 (IL-12), have shown therapeutic potential as antiviral and anticancer agents. This explains the activity of a PS-ODN antisense containing the CpG motif targeted to HPV, also showing activity for CMV and loss of activity in immune compromised mice [39]. This also explains the reason for flu-like symptoms with administration of GEM91, a PS-ODN antisense containing the CpG motif target to HIV-1 [55]. Interestingly, most of the PS-ODN antisense that were advanced to clinical development in the early 1990s contained unmethylated CpG motifs [51]. Thus, their mechanisms of action could be largely due to immune activation or side effects were caused by immune activation.

Detailed structure activity relationships have been carried to elucidate the interaction of PS-ODNs with TLR9. These studies have provided great insights. For example, (**a**) the presence of unmethylated CpG motif is required, although its position in the sequence is equally important [134], (**b**) accessibility of the 5′-end is required [111, 135], (**c**) modifications of the flanking sequence on the 5′-end impacts the immune activity [136], (**d**) methylation of C in the CpG motif neutralizes immune activation and causes it to act as an antagonist, and (**e**) certain modified bases could be used in the CpG motif without inducing immune responses. These insights have been very helpful in designing antisense candidates. These lessons have provided the basis for the creation of optimized agonists and antagonists of TLR9. These classes of compounds have been studied extensively in preclinical models of cancer [106], vaccines [137], viral infection [138], and autoimmune diseases [139], and clinical proof of concept has been established in multiple diseases [3]; Chapters 5 and 14 of Agrawal and Gait [3].

Detailed structure–activity relationship studies have been carried out for TLR3 [134], TLR7 and TLR8 [127, 140], RIG-I [141], and AIM2 [142]. It is important to take these insights into consideration when selecting a sequence and prioritizing chemical modification for use in therapeutic applications.

## 7 Conjugates and Delivery

The in vivo efficacy of ONs is defined by plasma half-life, tissue uptake, nucleolytic stability, and elimination. Systemic administration of several gapmer ASOs has shown a similar profile, i.e., short plasma half-life, wide tissue dispositions, and the presence of intact ASO for longer durations [121]. Even though the delivered gapmer ASO is present in targeted tissues including liver, for sustained clinical activity weekly dosing has been employed. This suggests that the administered ASO is not present in the right cells or cell compartment. Further insights came from intrathecal delivery of the 2′-*O*-methoxyethyl PS-ASO nusinersen (Spinraza) to treat SMA. Patients are being treated with IT delivery, administered only four to five times a year. This suggests that in a local compartment, a delivered ASO exerts pharmacodynamic activity for a longer duration and thereby requires less frequent dosing. In recent studies, both preclinical and clinical, conjugation of gapmer ASO with a GalNac cluster has been shown to improve potency and frequency of treatment for liver targeted RNA/gene targets. Efforts are being made to improve delivery of ASOs to muscles to treat muscular disorders employing antibody conjugates [143, 144].

Peptide conjugation has been researched extensively in recent years in efforts to increase the delivery of oligonucleotides. Numerous cell-penetrating peptides (CPPs) have been developed, which are beyond the scope of this Introduction. Readers are referred to a book describing methods that use cell-penetrating peptides [145]. However, the only peptides that have reached clinical development are Arginine-rich CPPs. These are not suitable for conjugation with negatively charged oligonucleotides because of the tendency of such conjugates to aggregate due to charge–charge interactions between the positively charged peptide part and the negatively charged oligonucleotide part. Instead they have found clinical utility for use as conjugates with charge-neutral PMOs. Here the company AVI Biopharma (now called Sarepta) developed a series of Arginine-rich CPPs that were taken to toxicological testing in monkeys but were found to have renal toxicity at elevated doses leading to a poor therapeutic index [146]. Recently Sarepta has advocated use of an alternative and shorter Arg-rich peptide, which is (Arg)_6_-Gly, as a PMO conjugate as a treatment for the neuromuscular disease DMD. This gave rise to significant improvements in delivery of an attached PMO and increased exon skipping [147]. The peptide-PMO conjugate is currently in Phase 2 clinical trials. Similar Arg-rich peptides, eg ones known as Pip having a short internal hydrophobic domain, have given rise to increased exon skipping for an attached PMO in muscles as well as in heart in an *mdx* mouse model of DMD [148]. Pip peptides and similar derivatives are currently being evaluated as potential therapeutics for other neuromuscular diseases, for example, in myotonic dystrophy [149]. Once again, shorter Arg peptide derivatives as PMO conjugates are likely to be the future clinical candidates in neuromuscular and neurodegenerative diseases.

Delivery of siRNA has been facilitated by the use of lipid complexes or as conjugates with GalNAc, mainly to the liver. Use of lipids provides stability to an siRNA candidate by encapsulating them, along with preferential delivery to the liver. Several lipid encapsulated siRNA candidates have advanced to clinical development. For example, patisiran formulated with lipid nanoparticles (LNP) has received regulatory approval. It is important to note that lipid-nucleic acid mixtures form complexes that create virus-like particle structures and engage PRRs to induce immune responses. In the case of patisiran, subjects were pre-treated with steroids to mitigate inflammatory responses.

The application of the use of GalNAc to hepatocytes has been known for some time and was employed for oligonucleotide delivery more than two decades ago [150]. GalNAc is a ligand for the asialoglycoprotein receptor 9 (ASGPR), which is very abundant on the surface of hepatocytes [151]. Conjugation with GalNAc generally leads to preferential delivery to the liver. However, depending on the nature of modifications of ONs, delivery to other compartments including the kidney has been observed. An siRNA-GalNAc conjugate givosiran has been approved for clinical use, another GalNAc conjugate. Inclisiran has shown positive results in a phase 3 clinical trial (Chapter 11 of Agrawal and Gait [3]).

## 8 Further Developments in Therapeutics

Over the years many other applications of nucleic acid-based therapeutics have been pursued. These include aptamers, CRISPR/Cas9, and use of modified mRNA for protein overexpression. While the construct and sequence of DNA or RNA employed in these uses may differ, one common aspect is the need to provide drug like properties to the selected agent. Most of the lessons learned in the development of chemistry in antisense field have facilitated the development of these approaches. In aptamers, modified nucleosides, such as 2′-*O*-methyl, 2′-fluoro or 2′-amino, and modified internucleotide linkages such as PS-linkages or borano-phosphate are regularly employed [152, 153]. In the case of mRNA therapy, considerations of use of chemical modifications are different than in other approaches. The 5′-cap and 3′-poly(A) tail are the key contributors to provide long half-life and for efficient translation. New capping agents such as 1,2-dithiodiphosphonate modified caps have been shown to improve RNA translation [154]. Several modified nucleosides including N^1^-methyl-pseudouridine and others have been useful in increasing efficiency of translation, and also mitigating immune stimulatory activity [155, 156]. The positional incorporation of modified bases in mRNA affects the secondary structure of the mRNA, which in turn influences its translation. Further stability to mRNA is provided by formulation with LNPs [157].

In studying CRISPR/Cas9-based therapeutic applications, several modifications are being evaluated. These include PS linkages and 2′-fluoro, LNA, c-Et [158], 2′-*O*-methyl [159], 2′,4′-BNA (NC) [N-Me] [160], etc. These chemical modifications not only provide stability but also mitigate interactions with PRRs. CRISPR-based technologies have been described in a recent book [161].

## 9 Summary

Nucleic acid-based therapies are now entering into their fifth decade (*see* Fig. 2 for a timeline of developments). Since the first report of the antisense principle in 1978 using unmodified ODNs, the technology has evolved, and drugs are now being approved. Based on the progress to date and the promise of the results, nucleic acid therapeutics are now being recognized as the third major drug discovery and development approach in addition to small molecules and protein/antibody approaches.

Nucleic acid therapeutic agents are built of A, C, G, T, and U nucleotides and connected through internucleotide bonds. Early work on chemical modifications to provide drug-like properties to antisense and lessons learned have been of tremendous value not only in creating antisense drugs but also in developing therapeutics using synthetic nucleic acids with other mechanisms of action (Fig. 4). Nucleic acid therapeutics could be broadly divided into two classes, the first in which an agent is created to target RNA or DNA and modulate its expression, and in the second an agent is created to bind to proteins or cellular factors. In both of these categories, agents could be recognized by PRRs thereby inducing immune responses, either unintended or intended affecting the mechanism of action.

The work on the chemistry of antisense has provided us with a few key modifications that have become important tools in nucleic acid therapeutics. The most important of these include PS linkages in ODN and ORN, gapmer design, selected 2′-*O*-sustituted nucleosides, and various bridged/locked nucleic acids, etc. The art of creating a nucleic acid agent lies in the understanding of putting together the nucleotide sequence and various modifications for its intended mechanism of action without interacting with PRR (Fig. 3).

## Figures and Tables

**Fig. 1:**
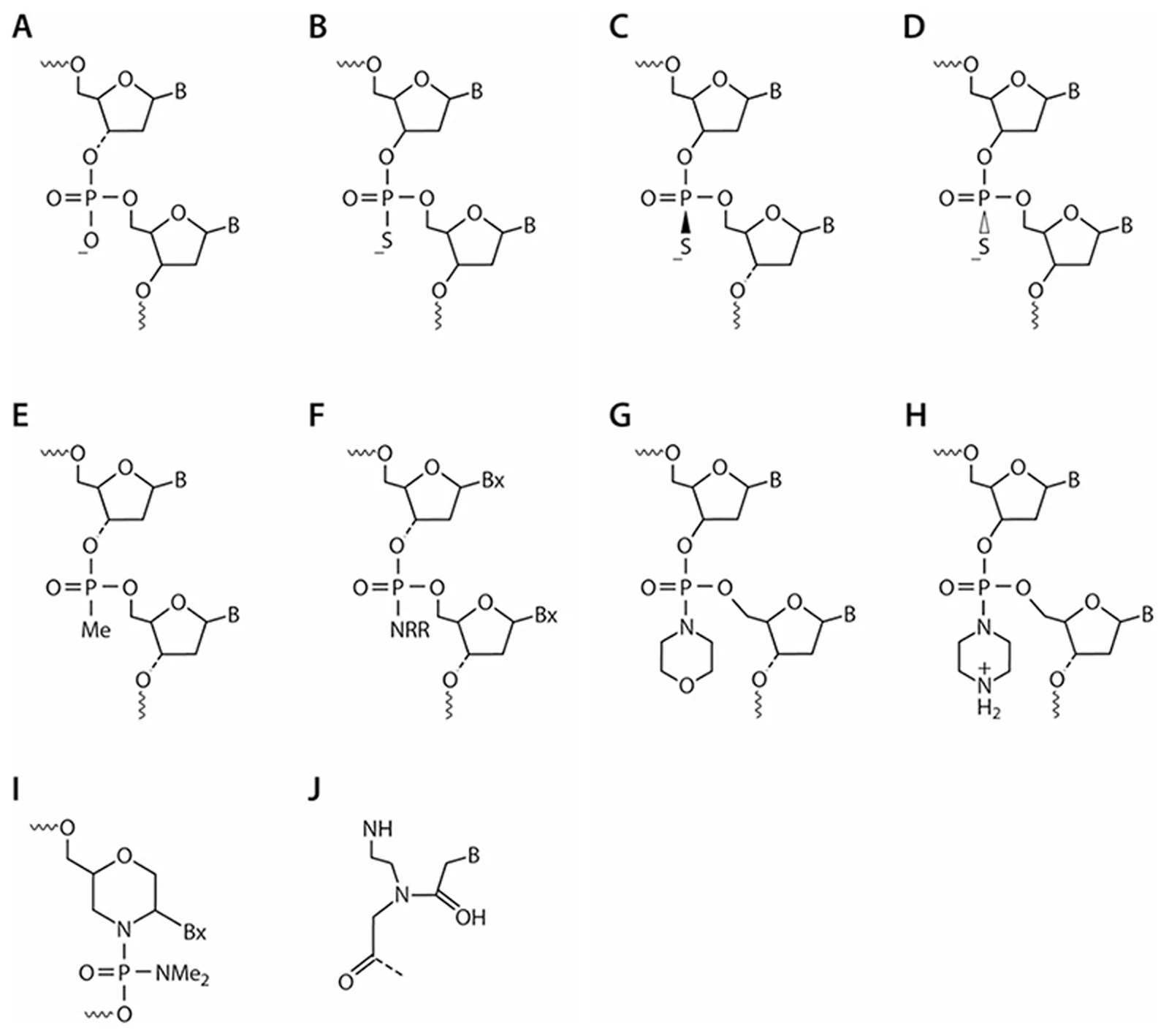
Chemical structures. (**a**) DNA oligonucleotides (ODN), (**b**) phosphorothioate ODN (PS-ODN), (**c**) *R*p-PS-ODN, (**d**) *S*p-PS-ODN, (**e**) methylphosphonate ODN (PM-ODN), (**f**) phosphoramidate ODN (PN-ODN) R = H or alkyl, (**g**) phosphomorpholidate, (**h**) phosphopiperazidate, (**i**) phosphorodiamidate morpholino (PMO), (**j**) peptide nucleic acids (PNA). B = heterocyclic base

**Fig. 2:**
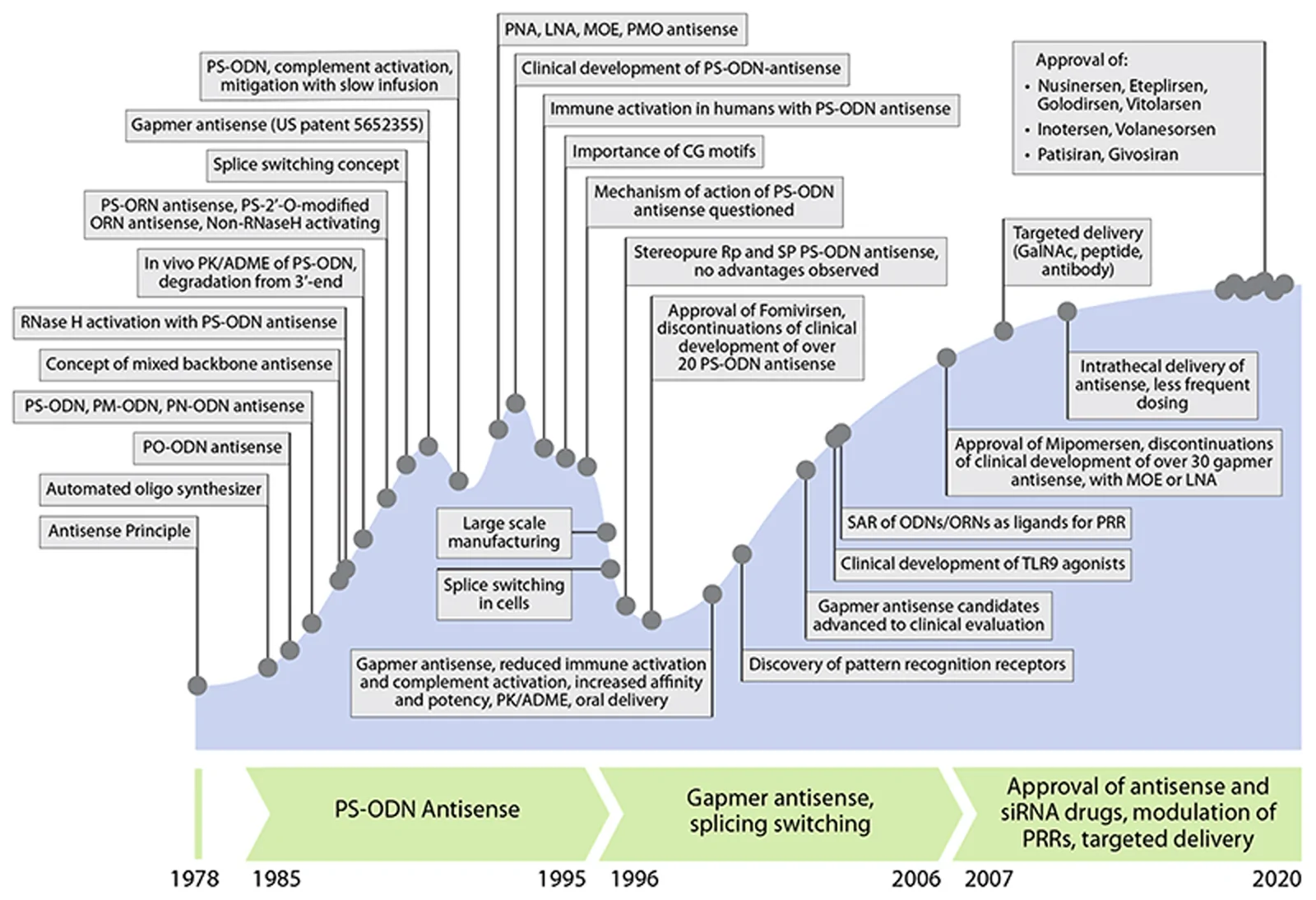
Evolution of chemical modifications during the development of antisense as therapeutic agents. In the nineties, various modifications of ODNs and ORNs were studied and based on RNase H activation, PS-ODNs became the choice as first-generation antisense agents. Soon it was realized that PS-ODN had off-target activities including complement activation and also sequence specific immune activation. This led to questions on the mechanism of action of PS-ODNs and associated safety signals, and clinical development of most of the PS-ODN ASOs was discontinued. In parallel use of modified ORN for splicing correction in the cells was established. Based on the early work conducted in early nineties, gapmer antisense design provided the key attributes and became the choice as second-generation antisense agents. Studies of chemical modifications in antisense has facilitated development of other therapeutic oligonucleotides. The key modifications which have been identified in the development of antisense, e.g. PS-PDN and PS-ORN, 2′-modified or 2′-*O*-substituted ribo-nucleosides, bridged ribo-nucleosides, and PMO which are being employed in various nucleic acid therapeutics. In the last few years, a number of drugs based on gapmer ONs (mipomersen, inotersen, volanesorsen), 2′-MOE PS-ORN (nusinersen), PMO (eteplirsen, golodirsen, vitolarsen), and siRNA (patisiran, givosiran) have been approved

**Fig. 3:**
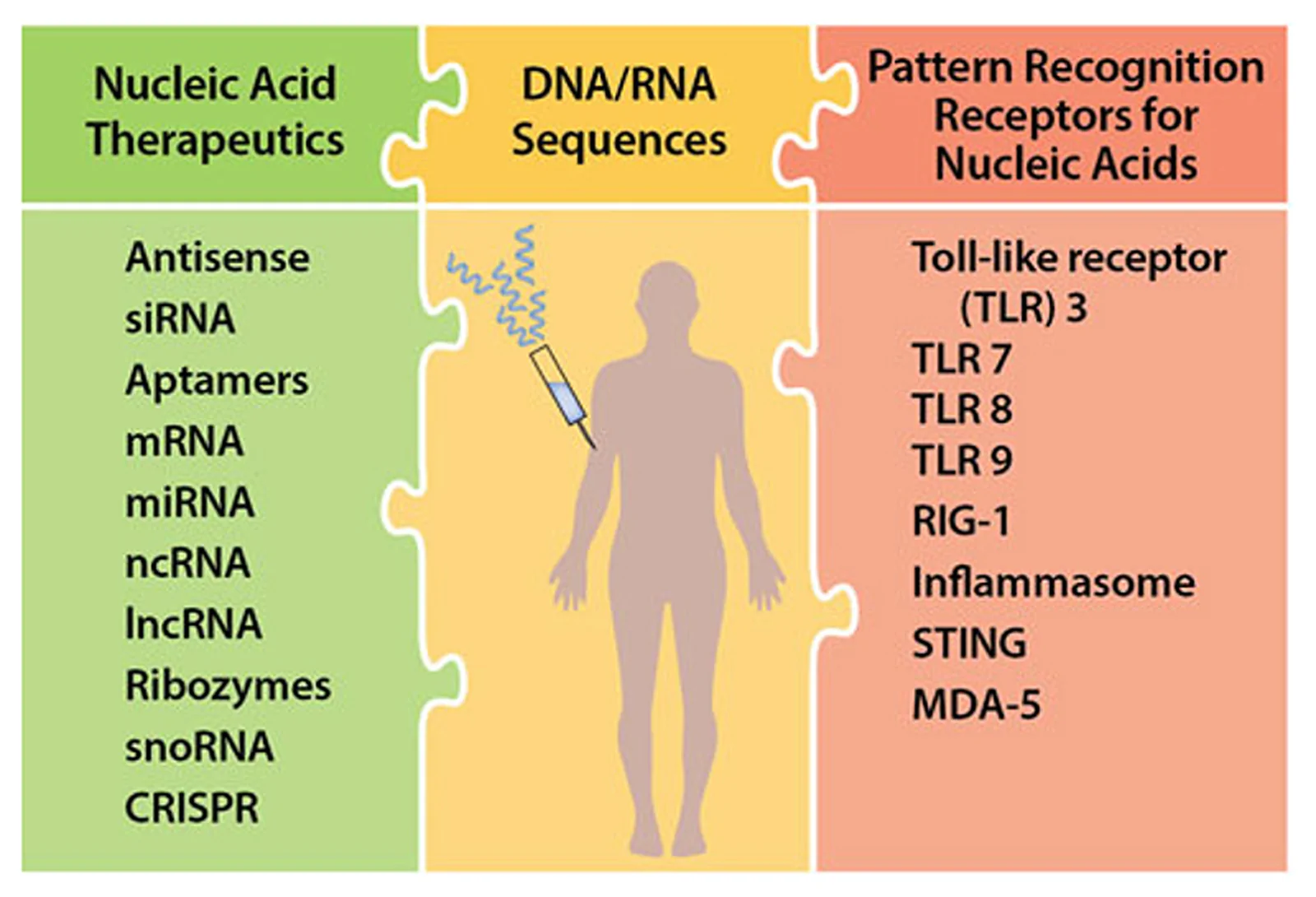
Pattern recognition receptors (PRRs). The number of PRRs listed in the right-side column which is known to recognize patterns of nucleic acids and induce appropriate immune responses. This recognition is applicable to all use of nucleic acids for therapeutics, and depending on the engagement could affect the mechanism of action and also safety signals. This recognition could be due to PAMPs of the nucleic acid agent being used or due to DAMPs, due to the build-up of administered agent in the tissues and organs, being recognized as endogenous nucleic acids

**Fig. 4:**
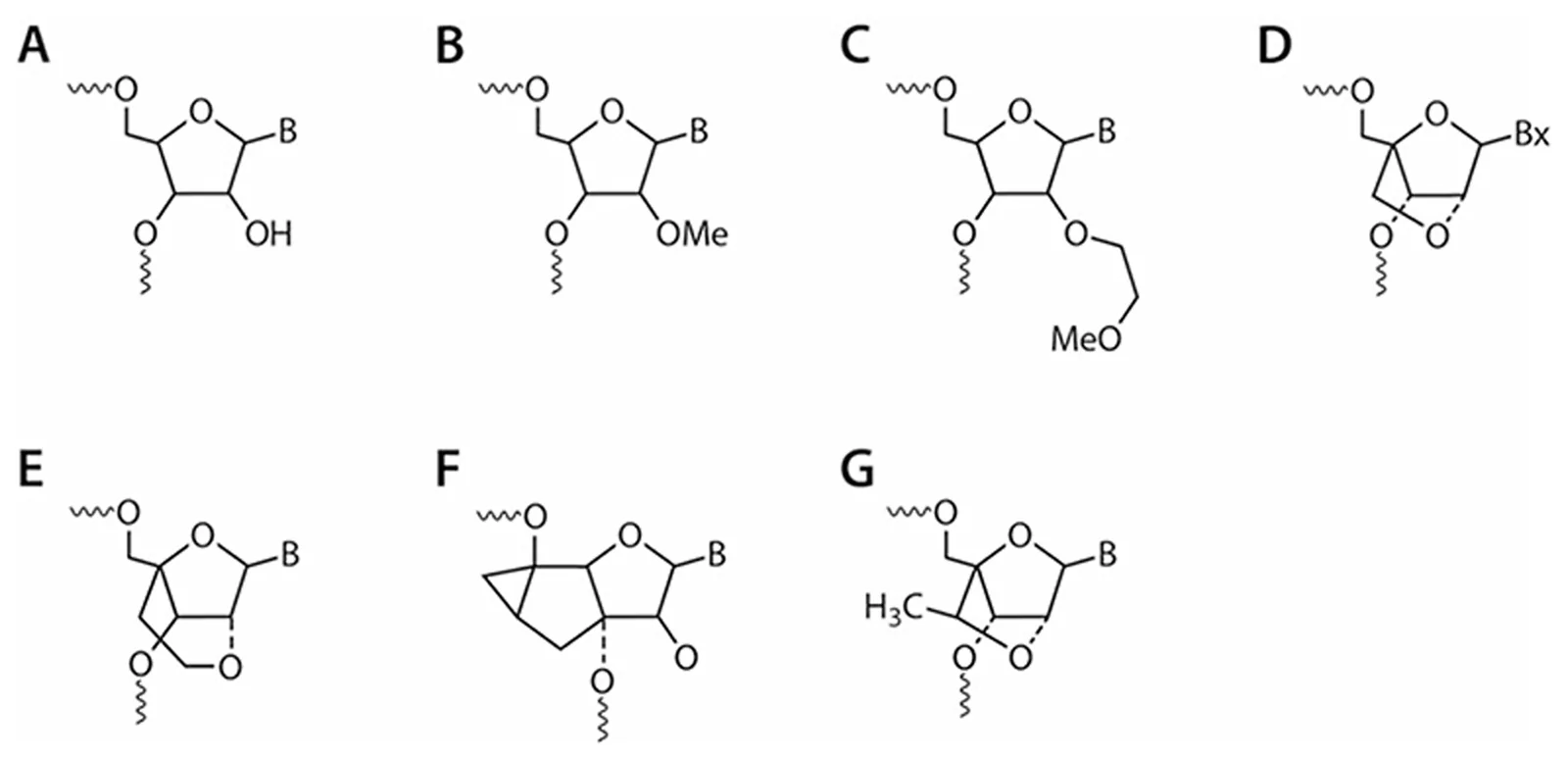
Chemical structures of the ribo-nucleoside units of therapeutically useful RNA and RNA analogues. (**a**) ribo-nucleoside (ORN) (**b**) 2′-*O*-methyl (2′-OMe), (**c**) 2′-*O*-methoxyethyl (2′-MOE), (**d**) bridged/locked nucleic acid (LNA), (**e**) 2′-*O*,4′-C-ethylene linked nucleic acid (ENA), (**f**) tricyclo-DNA (tcDNA), and (**g**) constrained ethyl (cET). B = heterocyclic bases

**Fig. 5:**
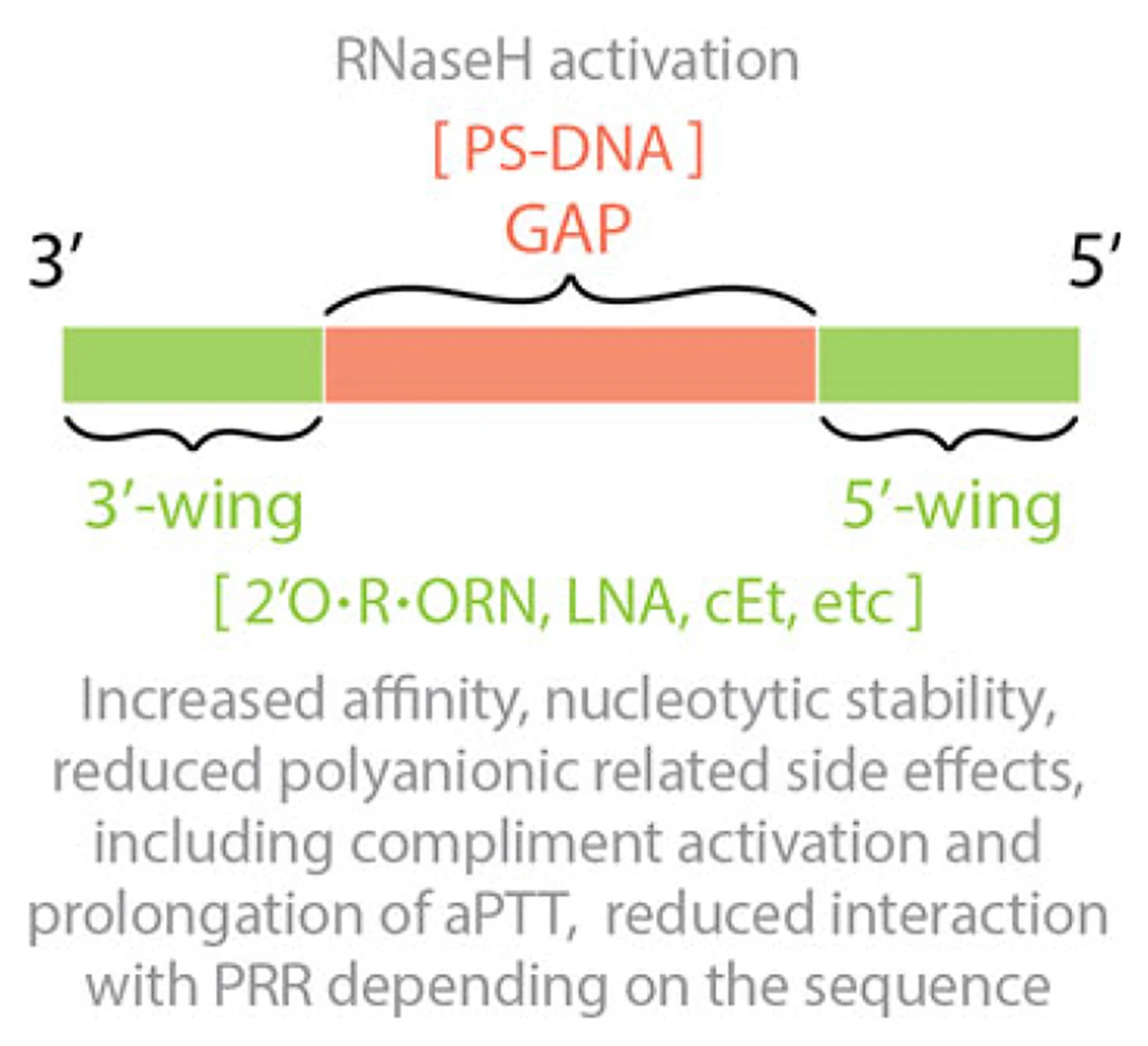
Design of gapmer antisense oligonucleotides. In a gapmer antisense, segments of PS-DNA and modified RNA are appropriately placed to combine desirable characteristics for antisense agent with both of these modifications. PS-DNA segment provides RNase H activation, and modified RNA segments provide increased nucleolytic stability, affinity to target RNA, decreased polyanionic characteristics and inflammatory responses
